# Effect of Intrinsic Stress on Structural and Optical Properties of Amorphous Si-Doped SnO_2_ Thin-Film

**DOI:** 10.3390/ma10010024

**Published:** 2017-01-01

**Authors:** Honglong Ning, Xianzhe Liu, Hongke Zhang, Zhiqiang Fang, Wei Cai, Jianqiu Chen, Rihui Yao, Miao Xu, Lei Wang, Linfeng Lan, Junbiao Peng, Xiaofeng Wang, Zichen Zhang

**Affiliations:** 1 Luminescent Materials and Devices, South China University of Technology, Guangzhou 510640, China; ninghl@scut.edu.cn (H.N.); lxz900618@icloud.com (X.L.); zhanghongkeke@gmail.com (H.Z.); fangzq1230@126.com (Z.F.); c.w01@mail.scut.edu.cn (W.C.); c.jianqiu@mail.scut.edu.cn (J.C.); xumiao4049@126.com (M.X.); mslwang@scut.edu.cn (L.W.); lanlinfeng@scut.edu.cn (L.L.); psjbpeng@scut.edu.cn (J.P.); 2 State Key Laboratory of Luminescence and Applications, Changchun Institute of Optics, Fine Mechanics and Physics, Changchun 130033, China; 3 Integrated System for Laser Applications Group, Institute of Microelectronics of Chinese Academy of Sciences, Beijing 100029, China; xiaofengwang@ime.ac.cn

**Keywords:** intrinsic stress, Si-doped SnO_2_, amorphous oxide semiconductors

## Abstract

The effect of intrinsic stress on the structure and physical properties of silicon-tin-oxide (STO) films have been investigated. Since a state of tensile stress is available in as-deposited films, the value of stress can be exponentially enhanced when the annealing temperature is increased. The tensile stress is able to not only suppress the crystallization and widen the optical band gap of STO films, but also reduce defects of STO films. In this report, the good electrical performance of STO thin-film transistors (TFTs) can be obtained when annealing temperature is 450 °C. This includes a value of saturation mobility that can be reached at 6.7 cm^2^/Vs, a ratio of I_on_/I_off_ as 7.34 × 107, a steep sub-threshold swing at 0.625 V/decade, and a low trap density of 7.96 × 10^11^ eV^−1^·cm^−2^, respectively.

## 1. Introduction

In recent years, the display industry is undergoing a significant shift from rigid displays to flexible displays. Transparent amorphous oxide semiconductors for thin-film transistors (TFTs) have been attracting tremendous attentions due to their low-temperature manufacturing process, highly optical transparency, good uniformity of film deposition and low cost [1]. Generally, interfacial defects of oxide TFTs between the channel layer and the dielectric layer can be reduced at high annealing temperature in the manufacturing process. Besides, it is important for oxide TFTs to be embedded in Polyimide (PI) substrate with tolerable temperature up to 450 °C for flexible displays [2,3]. However, in practical applications, the various stresses (i.e., tensile stress and compressive stress) of the oxide semiconductor films on plastic substrates may cause the fracture because the bending or stretching in plane strain can be initiated during the process of the large-area films production [4,5,6]. Therefore, avoiding the fracture caused by stress-related effects (i.e., keeping the integrity and stability of devices) is critical for flexible applications.

In general, all thin films can be considered in a state of stress during the deposition process, leading to different electrical and optical properties [7]. Therefore, the relationship between the stress of oxide semiconductors film and their properties should be systematically studied. In this study, STO film instead of the well-known indium-gallium-zinc-oxide (IGZO) film is investigated due to some merits of STO films, such as no toxicity, controllable components, and low cost. In fact, STO films not only exhibit an n-type oxide semiconductor but also own strong chemical stability, leading to the fact that they can be fabricated on back-channel-etched type TFTs [8]. Previously, Zhang et al. revealed that STO films related to doping element (Si and N) effects on heavily p-type silicon substrate [9,10]. For the flexible electronic device, we need enlarge the research on different substrates and stress situation.

In this paper, it is the first time at different annealing temperatures have been investigated. Finally, the optical band that characterizations for structural and optical evolution related to intrinsic stress of the STO films have been reported. In addition, the stress of such film used for fabricating device on the flexible substrate gap, surface morphology and oxygen vacancies correlated with the stress in the STO films have been studied respectively.

## 2. Experimental Methods

STO films are successively deposited on the surface of Si and glass substrate by radio frequency (RF) magnetron sputtering using an STO (SiO_2_:SnO_2_ = 5:95 wt %) target at room temperature. The power is 300 W, the ratio of Ar/O_2_ is 20:2 sccm with ambient pressure of 2 mTorr during the process. Finally, as-deposited STO films with thicknesses of 200 nm ought to be annealed at temperatures of 150, 250, 350 and 450 °C for 0.5 h on the hot plate, respectively.

**Figure 1:**
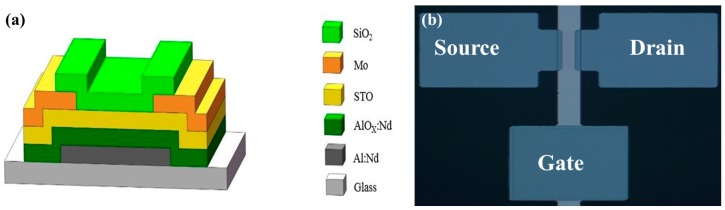
(**a**) Cross-sectional schematic of STO TFT; (**b**) Micrograph of STO TFT (Width/Length = 100 µm/40 µm).

To obtain STO TFTs with a bottom-gate inverted staggered-type structure (shown in Figure 1), a 300 nm Al-Nd alloy (3 wt % of Nd) as patterned gate metal can be firstly deposited by direct current (DC) magnetron sputtering. Subsequently, the film is anodized to form a 200 nm layer of AlO_x_:Nd on the surface in an electrolyte consisting of 3.68 wt % ammonium tartrate solution and ethylene glycol. Afterwards, a 5 nm STO thin film is deposited on the anodic oxide film by RF sputtering at room temperature. Then, for the source/drain (S/D) electrodes, a deposition of a metal film of Mo and a wet etched pattern have been processed in order to produce a channel with 100 μm width and 40 μm length when Phosphoric-Acetic-Nitric (70% Phosphoric acid, 10% Acetic acid, 5% Nitric acid) etchant is used. Finally, the post-annealing process of the device at 350 °C on a hot plate for 0.5 h is to be done before passivation of single-walled SiO_2_ films using plasma enhanced chemical vapor deposition (PECVD) at 250 °C.

The stress of STO films on Si substrate at different annealing temperatures has been characterized by the kSA Multi-beam Optical Sensor (MOS) system (KSA MOS Thermal Scan, k-Space Associates, Inc., Dexter, MI, USA). The structure and chemical state of STO films on glass substrate sare analyzed by X-ray diffraction (XRD) (Empyrean, PANalytical, Almelo, The Netherlands) and X-ray photoelectron spectroscopy (XPS) (Escalab 250 XI, Thermo Scientific, Waltham, MA, USA), respectively. The thicknesses of thin films are measured by a Dektak 150 surface profiler (Veeco, New York, NY, USA) and X-ray reflectivity (XRR). The surface morphology of films is obtained by atomic force microscopy (AFM) (XE-100, Eppendorf, Hamburg, Germany). The optical properties of the films are measured by a UV-3600 spectrophotometer (Shimadzu, Kyoto, Japan). The mobility and carrier concentration of the STO films are obtained by Hall-effect measurements (WAD (H.K) Co. Ltd., Hong Kong, China). The electrical characteristics of STO TFTs are examined in the dark using an Agilent 4155C semiconductor parameter analyzer (Agilent, Santa Clara, CA, USA) at room temperature.

## 3. Results and Discussion

**Figure 2:**
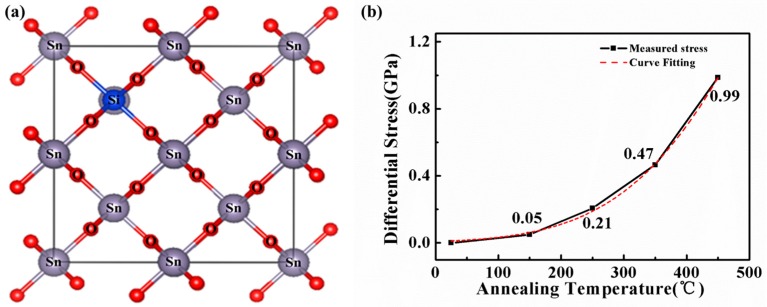
(**a**) The schematic structure of STO films; (**b**) The differential stress of STO films annealed at different temperatures. The red dash line represents an exponential curve fitting well with the experimental data.

**Figure 3:**
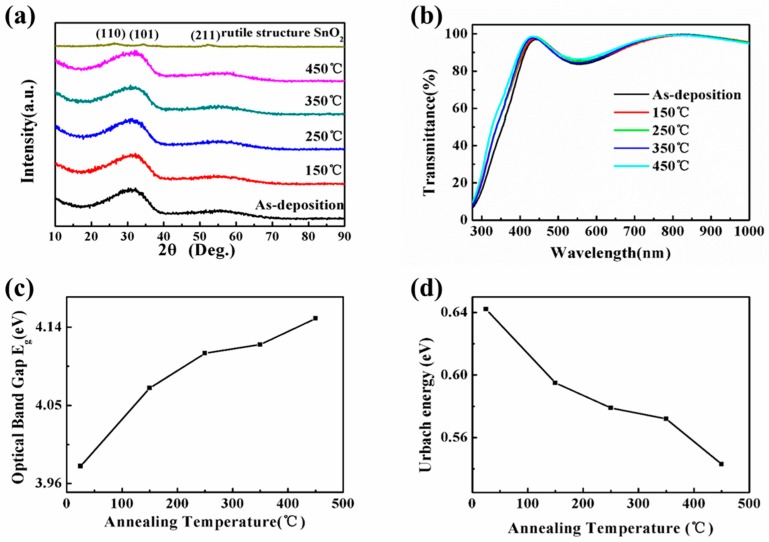
(**a**) XRD patterns; (**b**) Transmittance; (**c**) Band gap ($E_{g}$); and (**d**) Urbach energy ($E_{u}$) of 200 nm thick STO thin film annealed at different temperatures.

Generally, the stress of a film consists of intrinsic stress and extrinsic stress. The intrinsic stress is generated by the accumulating effect of defects because of the bombardment of the energetic species during deposition, while the extrinsic stress emerges due to lattice mismatch or different thermal expansion coefficients between the film and substrate materials. For all STO films, the extrinsic stress of such films can be avoidable because of excessive thickness [11], while their internal stress can be examined by comparing the radii of curvatures of the silicon substrates. Film stress (σ) is calculated according to Stoney’s Equation (1) [12]:

(1) $$\sigma =\left(\frac{\partial d}{d_{0}}\right)\frac{M_{s}h_{s}^{2}\cos\alpha }{12h_{f}L},$$

where $d_{0}$ is initial spacing, ∂d is the change in spacing, $M_{s}$ is biaxial modulus of the substrate, $h_{s}$ is the thickness of a substrate, $h_{f}$ is the thickness of a film, α is angle of incidence, and L is the distance from substrate to Camera CCD. In Figure 2b, the results of differential stress of STO films are proportional to annealing temperature from 25 to 450 °C. Moreover, good empirical fitting indicates that such STO films are in stretched states. The empirical fitting equation for STO thin films can be expressed below:

(2) $$\Delta \sigma =exp\left(-4.77206+0.01474T-9.26126\times 10^{-6}T^{2}\right),$$

where (Δσ) is differential stress, and *T* is annealing temperature. In order to understand the relationship between the structural properties of STO films and their corresponding stresses at different annealing temperatures, the XRD characterization is used. In Figure 3a, it is known that the SnO_2_ film exhibits typical rutile structure and the three peaks are at 26.6°, 33.8° and 51.8°, corresponding to the (110), (101) and (211) crystallographic planes, respectively. Notably, its crystalline structure has already been transformed into the amorphous structure when a small amount of Si infiltrates the SnO_2_ lattice (shown in Figure 2a). The STO films maintain their amorphous structure when annealing temperature is increased because the direction of crystallization of film is disordered by the increased tensile stress, resulting in the good uniformity over large areas and unchanged electrical properties. Figure 3b shows the transmittance spectra of STO films at different annealing temperatures in the wavelength ranging from 300 to 1000 nm. The average transmittance of all STO films is in the visible range of 80%. The optical band gap ($E_{g}$) can be calculated by the Tauc equation as follows:

(3) $${\left(ahv\right)}^{2}=A\left(hv-E_{g}\right),$$

where a is the absorption coefficient, h is the Planck’s constant, v is the frequency of theincidentphoton and A is the energy-independent constant [13]. The $E_{g}$ can be obtained by extrapolating the linear part of ${\left(ahv\right)}^{2}$ versus hv for all samples to the energy axis. The value of band gap ($E_{g}$) increases from 3.98 to 4.15 eV as the annealing temperature increased from 25 to 450 °C (Figure 3c). The change of $E_{g}$ can be mainly attributed to two factors: the local ordering and the stress. On one hand, atoms with thermal energy can be reordered in the STO films by the annealing process. This improves the local ordering and leads to the change of $E_{g}$. On the other hand, the repulsion between the oxygen 2p and the tin 5s bands is decreased when STO films are in stretched states [14]. As a result, the optical band gap is widened. Furthermore, there is a reduction for structural defects of the film at high annealing temperature (shown in Figure 3d). The Urbach energy ($E_{u}$) is an empirical parameter representing structural defects and can be extracted from the absorption coefficient [15,16]. The $E_{u}$ is extracted using Equation (4):

(4) $$\alpha =\alpha_{0}exp\left(\frac{hv}{E_{u}}\right),$$

where $\alpha_{0}$ is the pre-exponential factor. It is clearly observed that the value of $E_{u}$ displays a declining pattern.

In general, the intrinsic stress originated from the structural defects such as dislocations, nonstoichiometric components, and oxygen vacancies can be decreased when the annealing temperature is increased for a film. However, for STO films, the intrinsic stress is performed in a completely opposite manner due to two reasons: (1) the verification of the improvement of the tensile stress by increasing the density when the vertical thickness of STO films is shrunk (details are shown in Table 1). Moreover, the high tensile stress will further facilitate the carrier mobility due to greater overlapping of s-orbitals of the cations in STO films [17]; (2) the microstructure of the as-deposited STO films becomes more compact as the annealing temperature starts to increase. It is confirmed by surface morphologies of STO films using AFM characterization that the tensile stress is increased when the interaction of atoms is strengthened at annealing temperature of 250 and 450 °C.

**Figure 4:**
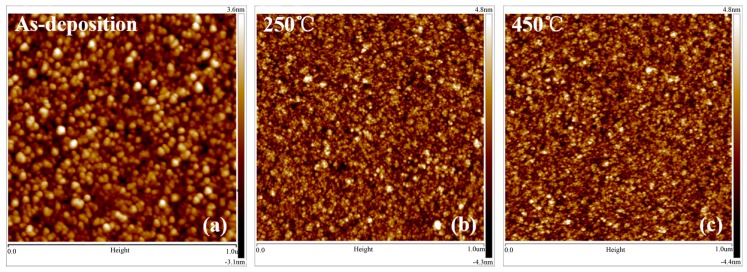
AFM images (1 × 1 μm) of STO films annealed at different temperatures: (**a**) As-deposition; (**b**) 250 °C and (**c**) 450 °C in air for 0.5 h.

In Figure 4, a large amount of round-shaped particle-like structures for the surface morphology of the as-deposited STO films starts to disappear when annealing temperature is increased up to 450 °C. Such behavior of formation and morphology for STO films is exhibited due to their dependence on interfacial energy [18]. It is also known that the defect states such as grain-like structure at low annealing temperature impacts the charge carriers’ transmission [19].

**Figure 5:**
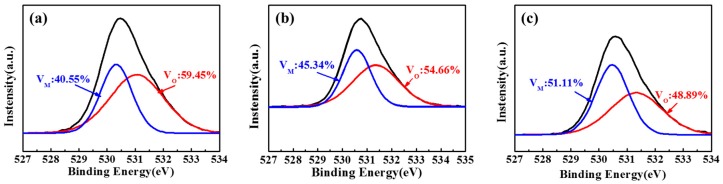
The O1 region of XPS spectra for STO films with different annealing temperatures: (**a**) As-deposition; (**b**) 250 °C; (**c**) 450 °C, respectively.

Figure 5 shows the XPS spectra of STO films in the O1 regions. The O1 peaks are divided into two principal peaks using a Gaussian–Lorentzian profile. The bind energy peak at 530.4 eV is attributed to M-O-M (V_M_) in the oxide lattices without oxygen defects, while the peak centered at 531.3 eV is assigned to the oxygen vacancies (V_O_). In general, the charge carriers in the oxide semiconductors are related to oxygen vacancies that donate two free electrons as shallow donors in STO films. The defects and the high carrier density of STO films will be suppressed at a high level state of tensile stress. The V_O_ of the STO films is decreased from 59.45% to 48.89% as the annealing temperature rises up to 450 °C.

**Figure 6:**
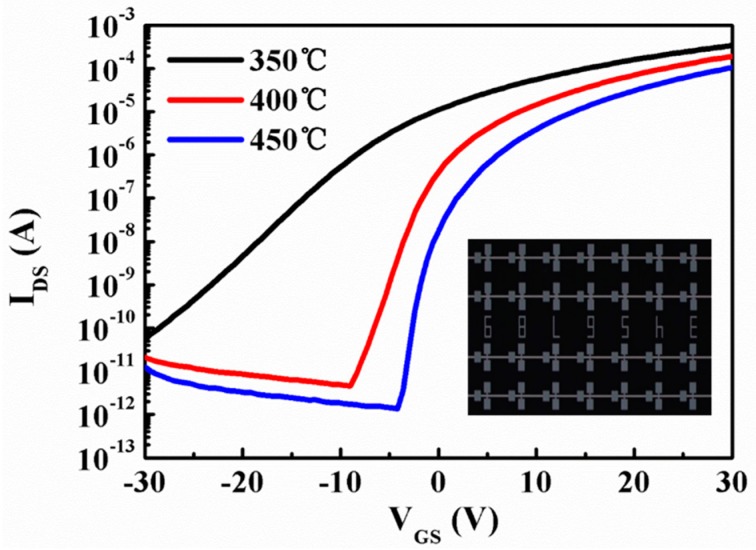
Transfer characteristic curves of STO TFTs annealed at 350, 400, and 450 °C, respectively.

In summary, STO TFTs is fabricated through optimized process. Figure 6 shows the transfer characteristic curves of STO TFTs with different annealing temperatures. The curves were measured at $V_{GS}$ = 30.1 V. The electrical performances of TFT are improved effectively by processing at different annealing temperature (shown in Table 2). The saturation mobility $\mu_{sat}$ is calculated by the following Equation (5) [20]:

(5) $$\mu_{sat}=\frac{{\left(\frac{d\sqrt{I_{DS}}}{dV_{GS}}\right)}^{2}}{\frac{1}{2}C_{i}\frac{W}{L}},$$

where $I_{DS}$ and $V_{GS}$ are the drain current and drain voltage, respectively. $C_{i}$ is the gate capacitance per unit area. The trap density ($D_{t}$) of the bulk states or the interface states between STO films and the dielectric interface can be calculated using the following Equation (6) [21]:

(6) $$D_{t}=\left[\frac{SSlog\left(e\right)}{\frac{k_{B}T}{q}}-1\right]\frac{C_{i}}{q},$$

where $k_{B}$ is Boltzmann constant, *T* is absolute temperature, $C_{i}$ is the capacitance per unit area, q is a unit charge, and SS is sub-threshold swing. As shown in Table 2, STO TFTs do not exhibit switching behavior at annealing temperature of 350 °C. However, when the device is annealed at 400 °C, the performances of $\mu_{sat}$, V_on_, I_on_/I_off_ ratio, SS and $D_{t}$ are 8.03 cm^2^/Vs, −9 V, 4.18 × 10^7^, 1.41 V/decade and 2.28 × 10^12^ eV^−1^·cm^−2^, respectively. When the annealing process is at a temperature of 450 °C, the performances of a $\mu_{sat}$, V_on_, I_on_/I_off_ ratio, SS and $D_{t}$ are 6.7 cm^2^/Vs, −4.8 V, 7.34 × 10^7^, 0.625 V/decade and 7.96 × 10^11^ eV^−1^·cm^−2^, respectively. From the above results, it is obviously observed that the oxygen vacancies and the trap states can be effectively reduced, and better electrical performances can be obtained at a high annealing temperature of 450 °C.

## 4. Conclusions

The relationship between the stress and optical band gap, the surface morphology and the oxygen vacancies of STO films at different annealing temperatures have been systematically investigated. It is found that STO films exhibit tensile stress, which increases exponentially with the rise of annealing temperature. In addition, the control of tensile stress of STO film is implemented by the annealing process. The tensile stress can suppress the crystallization and widen the optical band gap of STO films, and also minimize the defects in STO films. STO TFTs annealed at 450 °C exhibit better performance, with a saturation mobility of 6.7 cm^2^/Vs, a high I_on_/I_off_ ratio of 7.34 × 10^7^, a steep sub-threshold swing of 0.625 V/decade and a low trap density of 7.96 × 10^11^ eV^−1^·cm^−2^. These results suggest that the STO film is a promising active layer for transparent flexible displays.

## Figures and Tables

**Table 1:** The summary of Hall mobility, carrier density, and density for STO films annealed at different temperatures.

Annealing Temperature (°C)	Carrier Mobility (cm^2^/Vs)	Carrier Density (cm^−3^)	Density (g/cm^3^)	Roughness (nm)
As-deposition	4.70	4.09 × 10^18^	5.723	0.75
150	5.66	7.86 × 10^18^	5.750	0.18
250	3.32	5.76 × 10^19^	5.822	0.19
350	6.62	5.43 × 10^19^	6.076	0.09
450	6.53	1.72 × 10^19^	6.250	0.09

**Table 2:** Device parameters of STO TFTs.

Annealing Temperature (°C)	$\mu_{sat}$ (cm^2^/Vs)	V_on_ (V)	I_on_/I_off_	SS (V/decade)	$D_{t}$ (eV^−1^·cm^−2^)
350	--	<−30	--	--	--
400	8.03	−9	4.18 × 10^7^	1.41	2.28 × 10^12^
450	6.7	−4.8	7.34 × 10^7^	0.625	7.96 × 10^11^
